# How can researchers engage and co-develop care economy research partnerships? Insights from the Australian care service leaders

**DOI:** 10.3389/fpubh.2025.1698650

**Published:** 2025-11-24

**Authors:** J. De Nicola, I. Blackberry, C. Overy, C. Maylea

**Affiliations:** Care Economy Research Institute, La Trobe University, Melbourne, VIC, Australia

**Keywords:** care economy, collaboration, stakeholder engagement, research priorities, knowledge sharing

## Abstract

**Background:**

The care economy is among the fastest expanding sectors worldwide, worth globally over $11trillion. In Australia the health and social care sectors grew by over 50% employing over 13.8 million people in 2024. This study analyses sector perspectives of challenges and practical actions to support this explosive growth. The care economy supports health and wellbeing across the lifespan and is essential to both economic growth and social equity. In Australia, the demand for care services is growing, yet the sector still faces significant challenges, such as fragmented funding, workforce shortages, and limited collaboration between service providers, care participants and researchers. A gap remains in understanding what frontline organisations need from research, and how stronger, more effective collaborative research partnerships can be built and strengthened.

**Methods:**

This study used an interpretive-descriptive qualitative approach to explore the research priorities, collaboration needs, and barriers faced by care economy organisations across Australia. Between December 2024 and May 2025, semi-structured interviews were conducted via Zoom with 21 leaders from aged care, disability, health, and community services. All the participants were members of La Trobe University’s Care Economy Collaborative Network (CECN) or individuals who had expressed an interest in the work being conducted by Care Economy Research Institute (CERI) at La Trobe University. Thematic analysis was conducted using NVivo^®^ software.

**Results:**

Participants highlighted challenges such as workforce shortages, under investment in training, disjointed data systems, and funding models that often create competition instead of encouraging collaboration. They also highlighted clear inequalities depending on location, especially for rural and culturally diverse communities. There was strong interest in research that is practical, co-designed with services, and grounded in real-world needs. Participants said they need more support to get involved in research, more balanced partnerships, and a greater focus on research that can applied into practice.

**Conclusion:**

Organisations across the care economy face common challenges like workforce shortages, burnout, limited funding, and fragmented data systems. While there is strong interest in using research to improve services, many providers lack the time and resources to engage effectively. Collaboration is often hampered by competition for funding, siloed sectors and a disconnect between research and frontline needs. Rural and culturally diverse communities face extra barriers, highlighting the need for place-based approaches. Strengthening partnerships, investing in workforce development, and focusing on practical, co-designed research including people with lived experience will be critical to driving meaningful improvements across the care economy. This study synthesises perspectives across aged care, disability, community health, and policy, offering a novel contribution cross-sector map of shared bottlenecks rarely analysed together. We translate themes into system-level actions to support ongoing care-economy reform in Australia.

## 1 Introduction

The concept of a care economy, encompassing both the paid and unpaid activities that maintain individual’s health, wellbeing and dignity, has moved in the last decade from a largely academic discussion to a central concern of economic and social policy (1). Care is now regarded as essential social infrastructure, critical both to inclusive growth and to gender equity. Formal care services already constitute one of the world’s fastest-growing industries, worth an estimated US $648 billion in the United States alone, while unpaid care, provided largely by families, is valued at around 9 per cent of global GDP, or US $11 trillion (2, 3). In Australia, the health and social care sectors grew by over 50% employing over 13.8 million people in 2024. This study analyses sector perspectives of challenges and practical actions to support this explosive growth (1). In a recent report, it was estimated that investing in care services across 82 economies could create 280 million jobs by 2030 and a total of 299 million by 2035 most of them for women (4). This aligns with the International Labour Organization (ILO) 2024 resolution that calls for integrated care policies to ensure gender equality and universal access to care (5). These employment figures reveal both the scale of care-related labour and its chronic under-recognition in conventional economic metrics. As demographics change and evolving labour-market conditions and shifting family structures drive demand for care services, governments are grappling with how to expand provision while ensuring quality, sustainability and decent work (3).

Internationally, multilateral organisations, including the ILO, the World Bank and UN Women, now treat investment in care as a macroeconomic and social protection priority. A recent scoping review of 354 empirical studies published since 2018 found that scholarly attention to the care economy has risen steeply. However, research remains highly uneven with over 40% of studies originating in just three countries (United States, China and the United Kingdom), limited focus on migrant workers, men in caregiving, and the economic valuation of care is scarce (3). Most investigations are small scale, cross-sectional or descriptive in design (3). These gaps somewhat limit policymakers’ ability to quantify returns on care investment, forecast workforce requirements, or design evidence-based supports for diverse caregivers.

Across regions, governments and partners are piloting public private partnerships (PPPs) to treat care as investable social infrastructure under clear public regulation. Although Australia has formulated a National Care and Support Economy Strategy, comparative insights from regional initiatives are essential for an informed sector-wide response. In Asia and the Pacific, The Asia Foundation’s Roadmap highlights blended finance, employer supported care and digitised platforms (6). Malaysia’s 2023 Care Economy Dialogue calls for whole of society collaboration between government, business, civil society and universities. Trade policy is also being used to draw in private capital, with IACEPA Katalis showing how the Indonesia–Australia agreement can accelerate investment (7). In Latin America, UN Women documents a regional shift to rebuild economies around care through new policies, budgets and partnerships (8). These approaches highlight key foundational elements of a care economy framework that can drive positive change across the sector.

Australia sits at the forefront of the burgeoning care economy. The care economy plays a central role and continues to expand within the health care and social support workforce (9). Care and support industries already employ a significant share of the workforce and are projected to fuel job growth over the coming decade. A series of Royal Commissions, into aged care, disability services, mental health, and Defence Force suicide, have laid bare systemic fragmentation and workforce pressures. Neglect, workforce shortages, poor regulation and fragmented and unequal service delivery across care systems have been exposed by these commissions. In response, the Commonwealth released a Draft Care and Support Economy Strategy and accompanying Roadmap in 2023 (10), signaling a commitment to long-term reform in regulation, pricing, data standards and workforce wellbeing and positions Australia as an exemplar in rethinking the social care infrastructure and may provide useful lessons for other countries facing similar issues (11).

Australian labour-market analysis shows the care workforce has systematically higher casualisation (28% versus 19% economy-wide) and more multiple-job holding, complicating service continuity and quality (12). Australian data quality remains an ongoing issue, as the Australian government acknowledges fragmented data as a barrier to reform, committing to a sector-wide Aged Care Data and Digital Strategy 2024–2029 and the National Aged Care Data Asset (13, 14). This underscores the need for research–practice partnerships that address real workforce constraints.

It is well established that research takes time to be translated into practice (15). Given the demand and need for more timely innovation to be adopted in practice across the care sectors, research needs to be conducted with people with lived experience, and public private partnerships. Yet despite this policy momentum, remarkably little is known about how frontline providers experience these challenges or what kinds of research partnership they find most useful. Against this backdrop, the present study explores the research and collaboration needs articulated by care economy organisations. By grounding a research agenda in the realities of industry partners, the study aims to support a strategic, coordinated response to Australia’s care-economy reform agenda and to contribute fresh insights to the international literature on building equitable and sustainable care systems.

## 2 Materials and methods

### 2.1 Study aim and research questions

To address this gap, the present study adopted an interpretive-descriptive qualitative design to elicit the research priorities, evidence gaps and preferred modes of collaboration identified by care economy organisations. Specifically, we asked:

1. What challenges do care-economy organisations identify as most urgent for research?
2. What facilitators and barriers do industry partners perceive when engaging with academic researchers or other providers?
3. What would best support organisations to innovate and improve care quality in their contexts?

By mapping these needs, the study seeks to inform a co-ordinated research agenda that is responsive to industry realities, aligns with Australia’s Care and Support Economy Roadmap, and contributes to the global evidence base on effective, equitable and sustainable care systems.

### 2.2 Study design

This project addressed several key gaps in research and practice within Australia’s care economy. It responded to the need for coordinated, cross-sector collaboration in an industry characterised by fragmented service delivery and isolated organisations. The project recruited representatives from organisations within La Trobe University’s Care Economy Research Institute’s (CERI) and the Care Economy Collaborative Network (CECN). CERI was established in mid-2023 to break down the silos in the health and social care sectors and to develop a collaborative platform for the future design of services across the care economy. CERI aims to achieve this by developing care models based on evidence and trialled in the field, demonstrating scalability and replicability of care models, accelerating the adoption of technology, harnessing data to demonstrate the real-world impact of services and products and leveraging its members’ collective research and industry capacity and capability to advocate for systemic change. CERI established the CECN in an effort to allow for improved connection and collaboration between researchers and organisations involved in service delivery across the care economy. By gathering insights from diverse stakeholders in the CECN, a better understanding of research priorities, fostering more relevant and sector-specific research initiatives has resulted.

The study employed an interpretative-descriptive qualitative methodology to explore the research priorities and collaboration needs of key stakeholders in the Australian care economy. Semi-structured interviews were conducted to allow participants to share issues most relevant to their organisations to offer better support for industry-led research and knowledge sharing. This approach facilitated valuable insights and recommendations from experienced stakeholders in the care economy and produced actionable recommendations for CERI to implement. An inductive thematic analysis was used to analyse the interview transcripts with themes developed directly from the transcripts using NVivo software.

### 2.3 Setting and participants

The study was conducted online with participants located across Australia (see Table 1), including both rural and metropolitan areas. Participating organisations represented a mix of small, medium and large aged-care providers across public, private and not-for-profit sectors, offering both residential and community services. Participants were all members of the CECN or who had expressed an interest in the work being carried out by CERI. Eligible participants were leaders or decision makers in a wide range of sectors operating in the care economy. These included aged care, disability support, childcare, family services and community health. The interviews focused on the participants’ research priorities, ideas for improving collaboration, and the kinds of support they expect from the network. To gain a clearer understanding of the research priorities, collaboration needs, and expectations of key stakeholders in the care economy, email invitations were sent out to a variety of leaders and key role players across the care economy who had expressed an interest in the work conducted by CERI. Of the 87 original invitations sent, twenty-one participants were interviewed and were able to provide insights into the research and collaboration needs of their respective organisations. Recruitment was conducted via email and the interviews were conducted via Zoom. Following the interview, the participants were given the opportunity to review and edit their transcripts prior to the thematic analysis.

**Table 1:** Participants.

Participant	Care sector	Role	Location of interviewee	Size	Funding	Operating area
1	Public Health	Physiotherapist and director of organisation	Victoria	Small	For profit (FP)	National
2	Community Health	Chief operating officer	NE Victoria and Southern NSW	Medium	Not for profit (NFP)	Victoria (NE Victoria & southern NSW)
3	Aged-care assistive technology	Director research and development	Victoria	Medium	FP	Victoria
4	Public Health	Managing director	Victoria	Medium	FP	National
5	Public Health	Product portfolio manager	Victoria	Medium	FP	National
6	Disability	Chief operating officer	NSW	Medium	NFP	New South Wales
7	Community Health	Executive director	ACT	Small	NFP	National
8	Community-based education and workforce support	Care and Support Industry Practice Network Coordinator.	Victoria	Small	NFP	Victoria
9	Palliative care	Chief operating officer	ACT	Medium	NFP	National
10	Social infrastructure planning, inclusive design, and community wellbeing.	Lecturer in Social Design | School of Architecture, Design and Planning	NSW	Small	University	NSW
11	Child, family, and community welfare	Executive director of Education and Statewide Services	Victoria	Large	NFP	Victoria
12	Child, family, and community welfare	Executive director for People and Culture	Victoria	Large	NFP	Victoria
13	Family, child and community welfare	Senior manager	Victoria	Large	Government agency	Victoria
14	Aged Care	Gerontologist, lecturer	The National University of Malaysia	Medium	University	National
15	Aged care	Honorary Professor	NSW	Large	University	National
16	Community Health	Rural Nursing and Allied Health Research Fellow	Victoria	Large	University	Victoria
17	Disability	General manager of innovation and impact	Victoria	Medium	NFP	Victoria
18	Aged care	General manager	Victoria	Large	NFP	Victoria
19	Mental Health	Chief social worker	Victoria	Large	NFP	Victoria
20	Community mental health	Chair of the board	Victoria	Medium	NFP	National
21	Aged care	policy and research manager	Victoria	Medium	NFP	National

### 2.4 Interview procedure

A semi-structured interview guide was developed by the research team to gauge the participants’ research priorities, collaboration needs and expectations of working with researchers through networks such as the CECN. All the interviews were conducted via Zoom using the platforms cloud recording function. The interviews were all transcribed using the transcription software otter.ai and a verbatim transcription was sent to the participants for approval. Participants were encouraged to review and edit the transcriptions to ensure accuracy of the data prior to the thematic analysis.

### 2.5 Data analysis

We used interpretive-descriptive qualitative analysis to inductively develop themes across interviews. Analysis proceeded in three cycles: (1) open coding of an initial tranche of transcripts to surface candidate codes; (2) codebook development through memoing and collapsing overlapping codes; and (3) focused coding to elaborate themes, relationships, and disconfirming cases. NVivo (QSR International) was used for data management, memoing, and audit trails; software supported organisation, not interpretation.

One researcher (JDN) independently coded all transcripts. A second researcher (CM), reviewed all transcripts. The team then met regularly throughout data collection and analysis phases to compare interpretations, and resolved disagreements by negotiated consensus, updating code definitions with examples, iteratively checking fit and seeking counter-instances. We documented decision outcomes and used peer debriefing with a senior scholar (IB) and care economy leader (CO) not involved in data collection to challenge theme boundaries and naming. In line with reflexive thematic traditions, we emphasised interpretive consistency over mechanical reliability metrics; we therefore report on overarching themes and consensus issues rather than kappa coefficients.

The team comprised researchers with backgrounds in public health, social work, management and law, all affiliated with CERI, which may incline us toward system-level interpretations and implementation feasibility.

### 2.6 Ethics approval

Ethics approval for this study was granted by La Trobe University Human Research Ethics Committee (HEC24444). All the participants were provided with a Participation Information and Consent Form (PCIF) which was signed by them and returned to the research team. In the PCIF, the participants were informed that their information would be kept for five years, after which it would be securely destroyed. Participants were selected based on their capacity to inform strategies for improving research collaboration and knowledge sharing within the sector. Prior to the commencement of the interview, all participants were informed that that any information identifying them would be removed and that the transcription would be anonymised and confidential. At the time of the interview, verbal consent to proceed and to record the interview was received from the participants.

## 3 Results

Participants identified a series of interconnected challenges limiting the care economy’s capacity to engage in research, adopt innovation, and collaborate effectively. The findings are organised into thematic areas: funding and sustainability, workforce development and psychosocial risk, collaboration and competition, data and evidence systems, technology and innovation, equity and place-based concerns, and research priorities.

### 3.1 Funding and sustainability

Funding constraints and the lack of sustainable support structures were consistently identified as key barriers to research capacity, collaboration, workforce retention and long-term service delivery. Participants described how fragmented and inadequate funding limited their ability to engage meaningfully in partnerships and pursue strategic initiatives. Even when collaboration was mutually desired, strict guidelines and the absence of dedicated resources made it hard to move beyond initial conversations: “we always begin with ‘we should talk. We should find a way to work together’. That part is easy… The actual how do we do this gets tricky because of funding… but actually getting a fully funded project up off the ground can be quite challenging without dedicated funding sources.” #21.

Participants linked funding shortfalls with compromised care. A chief operating officer in palliative care observed, “People should not have to die alone. So that is increasingly happening more and more because of insufficient funding. There is insufficient access.” #9.

Complex and competitive funding models were said to create administrative burden, hinder long-term planning and encourage casualisation. As one executive director in child, family, and community welfare noted, “So, the funding tends to drive a more casualised kind of workforce.”#11.

Limited internal capacity further curtailed research participation. A care and support industry practice network coordinator said, “…trainers and center managers, they are drained. They’re exhausted.” #8 A chief social worker at a large urban hospital added, “…do not really have the capacity to have our own research priorities. Previously, we did have a research coordinator… but we have not got that.” #19.

Competitive processes were viewed as disadvantaging rural organisations. A rural nursing and allied health research fellow explained, “when you go to an open, competitive funding arrangement, I end up competing with metropolitan based researchers who… probably [have] a better track record… they’ll get the funding… over rural based researchers who are potentially emerging… but belong in the communities.” #16 Overall, participants viewed competitive funding structures as a system barrier to collaboration, undermining workforce retention, limiting research capacity, and compromised the quality service delivery across the care economy (18).

### 3.2 Workforce development and psychosocial risk

Workforce development and sustainability, and psychosocial risks were consistently identified as interconnected challenges. Participants described a sector under strain, with staff shortages, low retention, limited training opportunities, and unclear career pathways. These structural problems were compounded by the emotional demands of care work, including exposure to trauma, burnout, and insufficient formal support. A product portfolio manager in public health noted, “The system pushes people out because it’s too hard to stay in it for long.” #5

Training and workforce development were recurring themes. Participants highlighted the need to better prepare staff for complex needs. A physiotherapist and director stated, “What needs to improve is to have a better workforce skills development method…” #1 Another participant added, “People burn out quickly because there’s no clear path forward…” #6.

Government funding models and industrial relations settings were seen to encourage casualisation, undermining stability. An executive director explained, “So, we know, for example, that having a more permanent workforce delivers better quality outcomes but a lot of the way the care economy is structured and driven largely by government funding and government policies actually creates huge barriers to that. So, the funding tends to drive a more casualised kind of workforce.” #12 She also expressed frustration over cuts to training, despite recruitment initiatives: “So, on the one hand, we have the government reaching out to us with a number of initiatives about, you know, helping people move from other sectors into our sector, but then at the same time, they are cutting funding of TAFE qualifications.” #12.

Participants also spoke about the broader undervaluing of care work and the need to professionalise the sector. A policy and research manager in aged care put it plainly: “So that’s professionalization of the workforce, so better paying conditions, which will attract people to work in the care economy as a career… that people feel proud to work in… unfortunately, the care economy is characterized by low paid, low skill… But it’s not a job that is celebrated and respected exactly… what does that say about us as a society when we do not value those occupations and we do not reward them, and we do not view them as a particular skill set.” #21 Some organisations are responding by building capability: “We’re developing and co-designing training for staff across primary health.” #21

Participants agreed that day-to-day pressures and structural settings reinforce each other, leaving little space for long-term improvement in workforce development or psychosocial supports.

### 3.3 Collaboration drivers versus competitive barriers

Competition for funding and staff discouraged openness and joint efforts. A chief operating officer in community health said, “…we are also competitors, unfortunately, and it’s the way the system is set up, the funding bodies are set up that we are often competing for the same funding, or we might be competing for the same staff.” #2

Shared goals and aligned outcomes were seen to counteract this. A general manager of innovation and impact in the disability sector reflected, “…there are very similar issues in, say, aged care or in health workforce… what can we learn from that, and how can we input into… different ways of working. We are all facing similar issues… There is also… an interest… around systemic change.” #17

Structural separation prevented shared responses and limited knowledge transfer. The same general manager noted, “…everything is fairly siloed… network meetings are… only… disability… not always helpful… I… have worked across age, care and disability, so there are lots of parallels.” #17 A lecturer in social design added, “it’s like each silo, and they are not talking to each other… There is very poor knowledge translation from the research to practice… and implementation.” #10

Smaller and place-based organisations felt excluded. A physiotherapist and director said, “So, we are often invisible.” #1 A chief social worker in mental health observed, “…it’s not necessarily looking at our area of practice… we are… benefiting… academic institutions… but [it] may not directly benefit us… may not inform… our own practice.” #19 A rural nursing and allied health research fellow put it starkly, “They do not want to know what I think. They just want to judge me.” #16

Participants emphasised the need for active facilitation and informal platforms for connection. As the general manager of innovation and impact put it, “Just having a kind of a go to… not so much what’s written, but what do you hear? What’s the emerging stuff that’s going on?… it can only benefit the entire… care economy.” (18)

### 3.4 Data and evidence systems

Participants described fragmented, incompatible data systems that limit linkage, comparison, and use for planning and improvement. Disconnected platforms, duplicated records, and inconsistent reporting were seen to obstruct evidence generation and obscure service gaps. A chief operating officer in a community health organisation put it plainly: “Our data systems are in a mess in that even the government cannot really pinpoint exactly where the gaps are because there’s so many data sources that do not talk to each other, that it’s almost impossible to try and triangulate real need. It’s difficult stuff, and no one’s really got the solution to that.” #2

Some pointed to emerging reforms and pilots as partial answers, while noting their limits. The UK’s Care Data Matters roadmap was cited as a whole-of-system model, and Victoria’s Health Passport as a local test of improved sharing, yet interoperability and the digital divide remain unresolved.

Practical constraints compounded these issues. A gerontologist and lecturer involved in community wellbeing and social infrastructure planning highlighted storage and security costs over time: “Keeping the data is very expensive… for 20 to 30 years from now… that’s a very big challenge in that research.” #14

Siloed systems also undermined person-centred care. One participant observed, “Very few people understand what [person-centred care] is, and nobody tracks it… all the structures are there. They’re just not linked. And they are just not using a system.” #1 Internal fragmentation within organisations persisted despite consolidation efforts. An executive director of education and statewide services noted, “We’ve got three different client systems now that we have merged… each fit for purpose, what you need for NDIS is different to what you need for government contracts.” #11 Reporting burdens further diverted effort: “We have to report to multiple agencies and funders, and they all want slightly different things.” #6

Participants pointed to workable enablers, including common data elements across programs, single-entry reporting portals, agreed interoperability standards for safe exchange, and front-line dashboards that turn reporting data into tools for improvement.

### 3.5 Technology and innovation readiness

Advanced tools are transforming care delivery, but uptake remains uneven due to structural and resource constraints. Access and implementation vary across the sector, with limited resources, skills gaps, and a need for shared training and support. Digital access and skills were not uniform, for example among refugee or asylum seeker communities. A care and support industry practice network coordinator said, “Everyone has to complete their Certificate 3 in individual care and support online, and that’s really challenging… we act a bit like a family for them… we can make it at their own pace.” #8

Participants identified artificial intelligence as a way to reduce repetitive administrative and operational work, while noting a skills gap. A senior manager in a family, child and community welfare organisation explained, “Well, I mean the obvious one that I actually am focused on the moment trying to get better at is how to use AI better, just to get rid of all the incredible amount of labour-intensive routine work we do.” #13

Some organisations are already using digital platforms to enhance quality and coordination. A product portfolio manager described a personal health record designed to support person-centred care across settings: “At its core, [product] is a personal health record… so the patient’s health can be adequately managed, irrespective of where they are getting their health from… They have all the information they need.” #5

Frustration was expressed about reliance on imported systems and static formats that limit analysis and patient involvement. A managing director in public health said, “It really pixxxs me off that we have to import software from and pay the 10s and hundreds of millions to multinationals, typically North Americans companies who have a different health system to us… I do not think the SPDRs and the My Health Records of the world empower a patient… My Health Record is all PDFs anyway. So, you cannot really make any predictive analysis or measurements.” #4

Across interviews, participants saw both promise and constraint, citing uneven access, funding limitations, and limited system-level support as the main barriers to realising technology’s benefits.

### 3.6 Equity and place-based concerns (rural, culturally diverse stakeholders)

Disparities in service availability, particularly in rural areas and for marginalised groups, were a persistent concern. Participants called for targeted, place-based initiatives that reflect local contexts and workforce realities.

A rural nursing and allied health research fellow described how metropolitan institutions often dominate rural agendas, limiting relevance and translation: “The problem, though, is, when you come to rural spaces, we are limited in our competitiveness… metropolitan researchers are doing rural research, but not really having a good understanding or belonging in rural communities… not speaking to the right people, not actually being able to translate their research… not having a really good grasp of how things work in rural communities.” #16

Recruitment and retention pressures compounded these gaps. A community mental health board chair said, “I think the other aspect that we really struggle with here, more so than metro, is staffing… There are not enough registered nurses available to do that… just finding staff is really difficult…” #20

Participants also highlighted barriers for migrant and refugee women entering care roles, including limited English, digital access and training support. A care and support industry practice network coordinator noted, “…they might need extra help with their foundation skills. So, their language, numeracy literacy, employability or digital skills.” #8

### 3.7 Research priorities

Participants prioritised research that is practical, co-designed, and readily translated to frontline improvement, especially for person-centred care and workforce wellbeing. A physiotherapist and director set the tone: “So, for me, the research priority is, I would like more projects to use an implementation methodology for person centred care… because often they are an expert in their subject matter, but they are not an expert in person-centred care or behavior change.” #1 A policy and research manager in aged care emphasised the translation task, “We do not undertake research ourselves, per se, but we work with our research members, particularly focused on research and translation activities, trying to bridge that very long gap between production of research and implementation into practice.” #21.

Capacity constraints limited participation, particularly where organisations lacked protected roles or skills for research. A chief social worker explained, “We no longer have a research coordinator… I actually have very little capacity to do any of it.” #19

Workforce wellbeing was a recurring priority, including retention, stress, and professional development. An executive director in community health noted, “…our emphasis… is on workforce development, retention and wellbeing of our members.” #7

Technology-focused priorities centred on practical benefits, including digital inclusion and measurable outcomes. A general manager in aged care described work on safe technology use with older people: “…we wanted to really focus on their understanding of cyber safety and with scams… what they do to keep themselves safe online… and… awareness and utilisation of technology to support independent living.” #18 A product portfolio manager underscored the need to demonstrate impact, “I’d say being able to work with AI in a productive way across the patient as well as connecting the team of carers would be a huge benefit. And then measuring… this is the ROI… improvements in patient outcomes… funder outcomes… provider outcomes.” #5

Several participants also called for research informed by First Nations knowledge, including caring for Country and caring for people, to strengthen training and practice with First Nations communities.

## 4 Discussion

This is the first study to examine the research priorities, collaboration needs, and structural barriers identified by care economy organisations in Australia. There are several interconnected challenges within the Australian care economy, including workforce shortages, underinvestment in training, disjointed data systems, and funding models that often create competition rather than collaboration. Participants highlighted clear inequalities based on geographic location, particularly for rural and for diverse communities. There was a strong interest in research that is practical, co-designed with services, and grounded in real-world needs. Participants expressed a need for more support to get involved in research, more balanced partnerships, and a greater focus on research that can be applied in practice. The main findings of the study were that Australia’s care economy faces common challenges including workforce shortages, fragmented funding, and disjointed data systems, which limit service delivery and research engagement and collaboration between different sectors of the care economy. Rural and culturally diverse communities experience added barriers and they are often excluded from research agendas dominated by metropolitan institutions. Despite these barriers, organisations showed strong interest in practical, co-designed research that can be applied directly to practice. The findings build on existing research that shows the need for reforms that build a stronger workforce, improve research capacity, and support long-term, fair partnerships in order for the care economy to provide more sustainable and inclusive outcomes.

The findings arise from qualitative accounts of stakeholders operating in specific policy and service contexts. We interpret them as evidence-based propositions however, further testing with comparative designs, linked administrative data, and implementation trials is required.

### 4.1 Intersecting challenges in the care economy

Key stakeholders described a cluster of inter-related challenges. Workforce shortages fragmented and competitive funding arrangements, limited research capacity, and siloed data systems emerged as key barriers to sustainable service delivery and collaboration between the different care sectors, reflecting the findings of recent government enquires (16, 19, 20). As conceptual frameworks, such as those developed by the United Nations’ Economic and Social Commission for Asia and the Pacific, attest these challenges within the care economy are closely connected and tend to reinforce one another (21). A lack of workforce capacity limits organisations’ ability to take part in research or innovation. Fragmented funding makes it difficult to plan strategically, and disconnected data systems prevent learning and collaboration across the sector. These challenges are interlinked and reinforce one another: limited workforce capacity hinders research, fragmented funding limits planning, and siloed data blocks shared learning. This aligns with the Commonwealth’s plan to streamline aged care information flows through the Aged Care Data & Digital Strategy 2024–2029 (13). In the UK, the Government backed “Care Data Matters Roadmap,” is bringing together separate health and social care records to encourage better planning and use of resources within the National Health System. In Victoria, Australia, the pilot Health Passport program (led by Monash University) shows enhanced data sharing and communication between consumers and service providers but does not yet address issues such as interoperability with other systems and the digital divide (where consumers in regional, rural and remote areas of Australia are constrained by access to reliable internet services).

Clearer definitions and improved measurement of the care economy are important for recognising care work and guiding evidence-based policies (3). Evidence from care-home research–practice partnerships in England further indicates that this approach produces more usable knowledge and implementation traction (22).

### 4.2 Demand for co-designed, actionable research

Participants expressed a clear preference for research that is practical, co-designed and rapidly translatable to service improvement. They emphasised that evidence is most useful when developed collaboratively, with shared ownership over the research process and outputs. In Australia, the relatively new phenomenon of Research Translation Centres, often involving complex interchanges between universities and health services show initial improvements in streamlining processes but tend to focus on clinical themes and siloed projects (23). Rural, remote and culturally diverse communities face compounded inequities in workforce supply, service availability and digital infrastructure. These inequities further limit their ability to engage in meaningful research partnerships, access up-to-date information, and improve services in line with national reforms. This is further reinforced by a persistent rural–metro divide, where metropolitan researchers often assume responsibility for rural research agendas, limiting the leadership and voice of rural organisations in shaping evidence that reflects their own priorities and contexts.

### 4.3 Implications for reform and collaboration

The study shows that organisations offering services in the care community are eager for more meaningful, long-term collaboration with universities, governments, and across other sectors in the care economy. Participants emphasised the need for shared governance? models that enable joint planning, sharing of resources, and more integrated data systems. They stressed that collaboration should not be limited to short-term projects or one-off funding, but integrated into the system to promote lasting resilience and support ongoing innovation.

These findings are especially relevant to the care economy service providers and broader reform efforts. They suggest that lasting change will require more than just investment in services and infrastructure but it must also include support for the relationships, skills, and systems that allow organisations to work together, learn continuously, and adapt to local needs.

By focusing on the voices of front-line leaders, this study provides valuable evidence to guide reform. It highlights the real-world barriers care organisations face and the kind of research and collaboration they find most useful. These organisations are not just recipients of policy rather they are essential partners in shaping and delivering it. Listening to their insights will be crucial to building a care economy that is fair, effective, and able to meet the needs of all Australians.

In 2025, Australia launched its first Care Economy Cooperative Research Centre (CRC), a 10-year, AUD$129 million partnership bringing together universities, industry, government agencies, and community groups. The Care Economy CRC is described as the largest initiative of its kind in the world focused on health and social care, and it aims to transform Australia’s care services through research-driven innovation. Its programs concentrate on three interconnected priority areas – developing new care technologies, building data-driven solutions, and innovating in workforce training and models – all with the goal of improving care quality, productivity, and sustainability. This reflects a strategic recognition that improving the care system requires both social and technological innovation, underpinned by research evidence.

The La Trobe University-developed Care Data portal (24) seeks to increase the visibility of care-related data that is often hidden or difficult to access. It does so by providing a searchable, open directory of key metadata, including information on location, quality, and demographics. Such infrastructure can help reduce barriers to data sharing and is comparable to the *Catalogue of Social Care Data*, compiled by the London School of Economics in the UK (25).

The roundtable discussion on the care economy, held as part of the Australian Government’s broader Economic Reform agenda, brought together key stakeholders from health, disability, ageing, and social services to address pressing challenges and opportunities in the sector. Central to the dialogue was the Productivity Commission’s interim report, *Delivering Quality Care More Efficiently*, which outlines a reform blueprint aimed at improving care outcomes while enhancing system-wide efficiency (17). The report recommends aligning fragmented regulatory frameworks through a national screening and registration system for care workers, embedding collaborative commissioning across local health networks, and establishing a National Prevention Investment Framework to support long-term wellbeing (26). Participants at the roundtable—including ministers, policy experts, and care sector leaders—emphasised the need to rethink productivity in human services, shifting focus from service volume to quality and outcomes (27). Together, these initiatives signal a growing recognition that care is not just a cost but a strategic investment in Australia’s future prosperity.

### 4.4 Positioning within global discourse

International reviews depict a field grappling with the same pressures reported by participants here. The 2025 scoping review of 354 studies maps a rapidly expanding yet uneven evidence base, concentrated in a handful of countries. These patterns mirror this study’s accounts of workforce strain, fragmented systems and the struggle to generate practice-ready evidence (3). The ILO’s 2024 Resolution on decent work in the care economy consolidates the case for coordinated policy across recognition, redistribution and reward of care, and for strengthening paid care jobs. Participants’ calls for professionalisation, psychosocial risk mitigation and stable roles align closely with this agenda (5).

Our emphasis on partnership is consistent with international evidence that structural integration alone has struggled to shift outcomes in Scotland without strong local partnerships and data capability (28, 29). By contrast, Uruguay’s national care system illustrates a care-centred policy architecture with explicit measurement and cross-sector governance—offering concrete design lessons for Australia’s reform agenda (30). Participants supported deeper collaboration with industry, philanthropy and government to move beyond short grants and pilots, and saw PPPs as one option to share risk, mobilise capital and scale childcare, workforce development and digital platforms, provided quality, equity and fair work are safeguarded through transparent governance and data. These priorities align with international guidance that emphasises blended finance and employer-supported models within strong regulation.

### 4.5 Implications for policy and practice

The findings highlight several practical implications for advancing Australia’s care economy planning going forward. Participants identified that competitive tendering and short funding cycles actively discourage the kind of cross-organisational collaboration needed to address entrenched, system-wide issues. Sustained partnerships are difficult to build in an environment where organisations must compete for limited resources. Many participants noted the need for more balanced research partnerships which underscores the need to shift from researcher-led approaches to projects that are genuinely co-designed and co-owned. Such a shift would allow care organisations to shape research agendas in ways that align with their service priorities and community needs.

A major barrier to research engagement is the absence of protected time and dedicated roles within provider organisations. Even highly motivated services struggle to participate in research without structural support. Addressing this will require specific investment in building research roles and capacity within the care sector.

Care providers need opportunities to build research capacity within industry, while academic researchers must be supported to work more effectively and equitably with service organisations.

Participants highlighted that data and systems support must better integrate service delivery with research and evaluation priorities. When data systems are better aligned with service needs, providers can more easily access and use information to drive practical improvements in care.

Finally, to address regional disparities, resourcing regional hubs that combine research capacity-building with local workforce development would support more equitable research participation across rural and remote areas to reduce rural inequities.

### 4.6 Strengths and limitations

The study involved a diverse sample of care economy organisations varying in sector focus, geographic location, and organisational size and an interpretive-descriptive design that captured nuanced, experience-based insights. This diversity enabled the study to reflect a wide range of experiences and perspectives. The use of an interpretive-descriptive methodology further strengthened the research by allowing for the capture of rich, experience-based insights that highlight the complex realities of service delivery and collaboration within the care economy.

However, some limitations should be acknowledged. The convenient sample was drawn from a self-selected network which may over-represent research-engaged organisations potentially biasing the findings toward more research-active perspectives. This pathway efficiently accessed information-rich stakeholders but also introduces a risk of favourability bias toward participating organisations and advocates of care-economy reform. We did not attempt heterogeneity by sector, role seniority, service type, and geography, so front-line workers, small rural providers, culturally and linguistically diverse communities, and consumers/carers are comparatively under-represented. In addition, the reliance on virtual interviews could exclude providers with poor digital access. As such, our findings support analytic generalisation to concepts and mechanisms rather than statistical generalisation to populations. Perspectives from highly invested actors may amplify perceived system problems/solutions relative to less engaged or resource-constrained providers. We therefore treat the themes as directional insights that warrant corroboration in broader, more representative samples and comparative designs (e.g., maximum-variation or stratified sampling with independent recruitment).

Lastly, the findings reflect views at one time-point amid a rapidly evolving reform context. As the care economy in Australia continues to evolve, organisational priorities and challenges may shift accordingly.

## 5 Conclusion

While additional research is needed to further substantiate these results, our findings indicate that organisations across the care economy are facing a range of practical challenges including workforce shortages, workforce burnout, fragmented data systems and limited funding. Collaboration among service providers is impeded by competition for funding, siloing of care sectors, a disregard for frontline workers expertise especially in the rural areas and a mismatch between research and frontline realities. Rural and culturally diverse communities face additional barriers that require place-based responses. Moving forward, there is a clear opportunity to build stronger collaboration and partnerships between researchers and service providers, support workforce development, and focus on practical research that leads to evidence-based improvements in care.

## Data Availability

The original contributions presented in the study are included in the article/supplementary material, further inquiries can be directed to the corresponding author.
